# MAGMAS Inhibition Enhances Temozolomide Efficacy in Chemotherapy-Resistant Glioblastoma Models

**DOI:** 10.1158/2767-9764.CRC-25-0493

**Published:** 2026-06-09

**Authors:** Javier J. Lepe, Jennifer D. Tran, Naomi Lomeli, Vi P. Dang, Valerie C. Nguyen, Parthiban Chokkalingam, Sasmita Das, Bhaskar C. Das, Daniela A. Bota

**Affiliations:** 1 Department of Experimental Pathology and Laboratory Medicine, University of California, Irvine, Irvine, California.; 2 Department of Neurology, University of California, Irvine, Irvine, California.; 3 Chao Family Comprehensive Cancer Center, University of California, Irvine, Irvine, California.; 4 Department of Biological Sciences, California State University, San Bernardino, San Bernardino, California.; 5 Department of Anatomy and Neurobiology, University of California, Irvine, Irvine, California.; 6 Division of Drug and Biotherapeutic Discovery, School of Pharmacy and Pharmaceutical Sciences, University at Buffalo, The State University of New York (SUNY), Buffalo, New York.

## Abstract

Glioblastoma (GBM, isocitrate dehydrogenase wild-type grade 4 astrocytoma) is the most aggressive and common brain tumor, characterized by increased proliferation, invasiveness, mitochondrial-dependent changes, and necrosis. GBM recurrence is universal despite the standard-of-care treatment with maximal surgical resection, radiation, and temozolomide (TMZ). Most patients relapse 6 to 9 months following initial diagnosis, and the median survival after recurrence is less than a year. Therefore, effective therapeutic strategies are needed to overcome glioma resistance mechanisms and improve long-term outcomes for patients with GBM. Mitochondria-associated granulocyte macrophage colony-stimulating factor molecule (MAGMAS, *PAM16*) is a nuclear-encoded mitochondrial protein subunit of the translocase of the inner membrane 23 complex that functions as an essential regulator of protein trafficking into the mitochondrial matrix. We previously demonstrated that MAGMAS is overexpressed in GBM and that the small-molecule MAGMAS inhibitor BT9 reduces mitochondrial respiration and is cytotoxic to glioma cells *in vitro*. In this study, we investigated the role of MAGMAS in GBM biology and the effects of MAGMAS inhibition on TMZ-resistant glioma lines and patient-derived glioma stem-like cells. We observed elevated *PAM16* levels in recurrent GBM, chemoresistant glioma cells, and during metabolic switching processes. Concurrent treatment with BT9 and TMZ significantly increased cell death compared with either drug alone in all glioma lines, irrespective of their TMZ resistance status. Additionally, GBM cells constitutively expressing sh*PAM16* became sensitized to TMZ both *in vitro* and *in vivo* in an intracranial xenograft model. Our findings suggest that targeting MAGMAS holds promise as a novel, effective therapeutic strategy for GBM.

**Significance:**

This study finds a link between mitochondrial protein MAGMAS and chemotherapy resistance in glioma central nervous system cancers. These findings can help improve our understanding of how mitochondria play an important role in chemotherapy resistance mechanisms - highlighting MAGMAS as a target to help enhance the efficacy of TMZ in chemotherapy resistance cells

## Introduction

Glioblastoma (GBM) is the most common and aggressive primary brain tumor, with a dismal 5-year overall survival (OS) rate of 7.2% (1). Despite the treatment options upon diagnosis, including tumor resection, radiation, and temozolomide (TMZ) chemotherapy, recurrence is universal (2). TMZ is a DNA-alkylating chemotherapeutic agent that methylates purine bases of DNA (O^6^-guanine, N^7^-guanine, and N^3^-adenine). The cytotoxic activity of TMZ is mediated by methylation at O^6^-guanine residues. During DNA replication, thymine instead of cytosine is incorporated into the complementary strand opposite O^6^-guanine. Ultimately, this results in DNA damage and accumulation of double-stranded breaks, which culminates in cell-cycle arrest at G2/M, and apoptosis (3). In response to TMZ-induced DNA damage, glioma cells upregulate DNA repair pathways such as the O^6^-methylguanine-DNA methyltransferase (MGMT, direct repair) and, less commonly, the base excision repair or DNA mismatch repair pathways (4). MGMT promoter methylation has been associated with an increased response to TMZ and improved survival in patients with GBM (5). However, recent clinical studies demonstrate that MGMT has a limited value as a biomarker due to its relative prognostic value in predicting survival in newly diagnosed patients and the lack of direct targeting approaches (6). A phase II clinical trial combining TMZ with the MGMT inhibitor (O^6^-benzylguanine, O^6^-BG) in recurrent GBM demonstrated little to no survival benefit, suggesting there are additional mechanisms that may contribute to TMZ resistance in GBM (7). The most recent Food and Drug Administration (FDA)-approved therapy for GBM, tumor-treating fields (TTF, Optune), was shown to significantly increase OS in patients who received maintenance treatment with TTFs plus TMZ as compared with TMZ alone following completion of chemoradiotherapy (20.9 vs. 16 months; refs. 8, 9). Although potential and novel therapeutics are currently being investigated for GBM, TMZ remains the mainstay therapy for this disease, and the development of combination therapies that can synergize with TMZ requires further investigation.

Mitochondria play a critical role in the development of TMZ resistance through multiple mechanisms, including metabolic reprogramming, regulation of apoptosis, increased cellular proliferation, mitochondrial biogenesis, and alterations in reactive oxygen species (ROS) production (10). We demonstrated that MAGMAS is overexpressed in GBM and plays a crucial role in glioma cell survival (11). Mitochondria-associated granulocyte macrophage colony-stimulating factor molecule (MAGMAS) is also overexpressed in metabolically active tissues (brain, skeletal, and cardiac muscles) and in other cancers, including breast, prostate, ovarian, and pituitary adenomas (12–15). MAGMAS is an inner mitochondrial membrane protein subunit of the translocase of the inner membrane 23 (TIM23) complex, which regulates the import of nuclear-encoded mitochondrial proteins into the matrix (16, 17). In addition to its role in mitochondrial protein trafficking, MAGMAS has been shown to increase cell tolerance to oxidative stress by modulating ROS production, enhancing antioxidant scavenging activity, and improving the activity of the electron transport chain (ETC) complexes (18). The various roles of MAGMAS in modulating cellular metabolism and mitochondrial protein import make MAGMAS inhibition a compelling strategy for targeting mitochondrial-mediated resistance mechanisms in GBM and improving the prognosis of patients with GBM.

BT9 is a novel, brain-penetrant, small-molecule MAGMAS inhibitor, which we previously reported to significantly induce mitochondrial dysfunction, intracellular vacuole formation, and apoptosis and decrease the proliferation of malignant glioma and medulloblastoma lines *in vitro* (11, 19, 20). Additionally, *in vitro* studies of BT9 in non–central nervous system (CNS) cancers, such as ovarian, prostate, and breast cancers, have shown that BT9 exerts cytotoxicity in differentiated cancer cells (11–13, 15). In this study, we investigated the role of MAGMAS in GBM biology and its involvement in TMZ resistance. We used genetic and pharmacologic approaches to modulate MAGMAS expression in glioma models *in vitro* and an orthotopic malignant glioma xenograft mouse model.

## Materials and Methods

### Cell culture

Patient-derived primary cell line DB93 was isolated from a GBM surgical specimen in the laboratory of D.A. Bota, in accordance with UC Irvine Institutional Review Board (IRB)-approved protocols (21, 22). GBM patient-derived xenograft (PDX) lines G12 and G85 were purchased from Mayo Clinic and maintained using the established Mayo Clinic protocols. The established human anaplastic astrocytoma line HOG (RRID: CVCL_D354) and patient-derived lines (DB93, G12, and G85) were maintained in glioma stem-like cell (GSC) media, Neurobasal Media without phenol red (Gibco), and supplemented with 1× B27 without vitamin A (Gibco), 1× GlutaMax (Gibco), 1× sodium pyruvate (Gibco), 1% penicillin/streptomycin (Gibco), amphotericin B (Corning), 20 ng/mL bFGF (Peprotech), and 20 ng/mL EGF (Peprotech). HOG cells were initially reported as oligodendroglioma; however, studies show that it lacks IDH mutations and does not have 1p/19q codeletions (23, 24). The other established human glioma lines D-54 MG (A172 derivative; RRID: CVCL_5735), U-251 MG (RRID: CVCL_0021), T98G (RRID: CVCL_0556), and HEK293T (RRID: CVCL_0063) cells were maintained in DMEM/F-12 (Corning) containing 292 μg/mL L-glutamine, with 10% FBS (Sigma) and 1% penicillin/streptomycin (Gibco). All cells were maintained at 5% CO_2_ at 37°C in a humidified incubator. MGMT methylation statuses of cell lines used are listed in Supplementary Table S1. Normal human astrocytes (HA, ScienCell, cat. #1800) were cultured in EGM-2 Endothelial Cell Growth Medium-2 BulletKit (Lonza). These cells were a gift from Christopher Hughes’s laboratory. All cell lines were tested for *Mycoplasma* and authenticated by DNA short tandem repeat profiling.

The study was conducted in accordance with the Declaration of Helsinki and approved by the IRB of the University of California, Irvine (UCI IRB HS#2012-8912) for studies involving patient-derived tumor specimens. Written informed consent was obtained from all subjects who donated tumors for the cell lines involved in the study.

### Generation of TR and OTR resistant cell lines

The U-251 MG and D-54 MG chemoresistant cell lines that were resistant to TMZ alone (D-54 MG-TR and U-251 MG-TR) or TMZ + O^6^-benzylguanine (D-54 MG-OTR and U-251 MG-OTR) were maintained using the established glioma cell line media as described above. For maintenance of TR and OTR statuses, the TR lines underwent treatment every three passages with 1,000 μmol/L TMZ (TCI) for 24 hours. The OTR cell lines were exposed to 1,000 μmol/L TMZ + 1,000 μmol/L O^6^-benzylguanine (O6-BG, Sigma). Parental TMZ-sensitive cells (U-251 MG S and D-54 MG S) received an equivalent dose of DMSO (Sigma).

### Drug treatment and cell viability

Compounds, except for sodium dichloroacetate (DCA), were solubilized with DMSO and further diluted in complete media for treatment. For XTT and MTT cell viability assays, 5 × 10^2^ to 1 × 10^4^ cells were seeded in a 96-well plate overnight and treated the following day. Cell viability following BT9 treatment was tested at 3 days, whereas TMZ viability experiments were done at 5 or 7 day time points. Synergy experiments were done at 3 days. Cell viability was assessed using the MTT assay for adherent cells, and XTT (Biotium) was used for cells (DB93, G12, G85, and HOG). Cells with MTT were incubated for 4 hours. After incubation, the supernatant was carefully aspirated, and formazan crystals were dissolved with 150 μL DMSO. Absorbance was read at 570 nm. Cells treated with XTT were incubated for 4 or 8 hours, and absorbance was read at 475 and 660 nm for nonspecific reads according to the manufacturer’s protocol (Biotium). Synergy analyses were performed using Synergy Finder+ software.

### Dedifferentiation of GBM cells in GSC media

GBM cell lines T98G, U-251 MG, and D-54 MG were dedifferentiated over 30 days by maintaining them in complete GSC media as described above.

### Quantitative PCR

Total RNA was extracted using an RNA extraction kit (Zymol) according to the manufacturer’s instructions. RNA quantification was performed using a NanoDrop 2000 (Thermo Fisher Scientific). A measure of 250 to 1,000 ng of RNA was used for cDNA synthesis using First-Strand cDNA Synthesis Kit (Quantabio). Quantitative RT-PCR was performed using PowerUp SYBR Green Master Mix (Applied Biosystems) and analyzed using the Quanti-Studio 6 PCR system (Applied Biosystems). The primers used in this study are listed in Supplementary Table S2.

### Mitochondria isolation

Mitochondria were extracted using previously published methods (25). Briefly, cells were harvested by scraping and washed twice with ice-cold PBS. Cells were then incubated with homogenizing buffer (30 mmol/L Tris-HCl, 225 mmol/L mannitol, 75 mmol/L sucrose, 0.05 mmol/L EDTA, and protease/phosphatase inhibitors) for at least 15 minutes and homogenized using a 1-mL syringe with a 25-gauge needle on ice. Samples were washed once at 7,000 and then 10,000 × *g*’*s* in buffer without EDTA. Mitochondria were lysed using RIPA buffer (Thermo Fisher Scientific) containing inhibitors for 10 minutes on ice and centrifuged at 15,000 × *g*’*s* for 10 minutes at 4°C. Samples were stored at −80°C until further use. Protein quantification was done using bicinchoninic acid (Pierce Protein Assay Kit).

### Western blot analysis

Protein lysates were run on a Tris-Glycine SDS-PAGE gel in SDS running buffer for ∼1.5 hours using a Mini Trans-Blot Cell (Bio-Rad). Proteins were then transferred onto a 0.2-μmol/L PVDF membrane overnight at 4°C. The membrane was then blocked with 5% milk in PBS-Tween 20 (PBST, pH 7.4) for 1 hour at room temperature (RT). Membranes were incubated with primary antibodies diluted in 5% BSA in PBST overnight on a slow rocker at 4°C. Membranes were washed three times with PBST for 10 minutes each and then incubated with secondary antibody for 1 hour at RT in 5% milk in PBST. Afterward, the membranes were washed three times with PBST and incubated with ECL chemiluminescent substrate (AZURE) for 2 minutes before imaging. For membranes that were reblotted with multiple antibodies, these membranes were incubated in stripping buffer (Thermo Fisher Scientific) for at least 15 minutes at RT, washed two times with PBST for 5 minutes and blocked again for 1 hour. To test whether previous antibody was completely removed, the blots were incubated again with the same secondary antibody and visualized for verification. Antibodies used in this study include the following: *primary* antibodies anti-MAGMAS (Proteintech cat. #15321-1-AP, RRID: AB_2878126), anti-TIMM23 (ABclonal cat. #A8688, RRID: AB_2772611), anti–superoxide dismutase 2 (SOD2; Abcam cat. #ab13533, RRID: AB_300434), anti–aconitase 2 (ACO2; Abcam cat. #ab71440, RRID: AB_1267614), anti–cytochrome c (Proteintech cat. #10993-1-AP, RRID: AB_2090467), anti-COXIV (Abcam cat. #ab62164, RRID: AB_2085277), anti-LONP1 (Proteintech cat. #15440-1-AP, RRID: AB_2137152), anti-mtND1 (ABclonal cat. #A17967, RRID: AB_2861769), anti-SOD2 (Abcam cat. #AB13533, RRID: AB_300434) anti–nuclear respiratory factor 1 (NRF1; ABclonal cat. #A5547, RRID: AB_2766328), anti-MGMT (Proteintech cat. #17195-1-AP, RRID: AB_2143221), and anti–Β-actin (ABclonal cat. #AC006, RRID: AB_2768236) and *secondary* antibody horseradish peroxidase–conjugated goat anti-rabbit (Millipore cat. #AP132P, RRID: AB_90264).

### ROS measurement

ROS analyses were performed using MitoSOX Red or CellROX Deep red reagent (Thermo Fisher Scientific). A total of1 × 10^5^ cells were seeded into a 12-well plate (*n* = 3 or *n* = 4) for each group. The following day, cells were treated with indicated doses of BT9 and N-acetyl-L-cysteine (NAC). After 24 hours, a final concentration of 2 μmol/L MitoSOX or 5 μmol/L CellROX Deep Red was added to the cells, and plates were incubated for 30 minutes at 37°C and 5% CO_2_. Cells were washed once with warm PBS and trypsinized for 3 minutes. Cells were collected then washed again with warm PBS and resuspended in ice-cold FACS buffer (PBS + 0.5% BSA + 0.05% sodium azide) and analyzed by flow cytometry (BD Fortessa). Median fluorescence intensity was quantified per sample.

### Annexin V–FITC and propidium iodide apoptosis assay

A total of 5 × 10^5^ U-251 MG cells were seeded in a 10-cm plate and treated with BT9 IC_50_ dose in combination with 2.5 mmol/L NAC or NAC alone for 3 days. A total of 1 × 10^5^ cells were stained with Annexin V–FITC and propidium iodide according to the manufacturer’s protocol (Proteintech). Samples were analyzed using a flow cytometer (BD Fortessa) to assess both early and late apoptotic cells.

### Generation of transgenic PAM16 knockdown glioma lines

Cells were seeded in DMEM/F-12 complete or GSC media overnight, and on the following day, the media were exchanged with media containing 8 μg/mL Polybrene. Generated viral particles (OriGene TL317164 and TR30031) were added, and cells were incubated overnight. The following day, the media were removed, cells were washed once with PBS, and the media were exchanged with fresh complete media for expansion. Successful transduction was observed by a positive GFP signal. Cells were subjected to clonal selection by seeding 25 cells into a 10-cm plate and allowed to expand stable single-cell colonies for extraction. *PAM16* knockdown was verified by Western blotting of mitochondrial lysates.

### TTFs

Glass cover slips were treated with 0.1 mg/mL of Poly-L-Lysine (Biomatrix) and washed with sterile water. Coverslips were placed inside the Inovitro ceramic well and loaded with 2 × 10^4^ cells suspended in 400 μL media for at least 1 hour until the cells attached. Then, 1 mL complete media was added to each well and placed inside the incubator overnight. For control cells not exposed to TTFs, cells were seeded at the same density in a 12-well plate. The next day, the media were exchanged with 2 mL complete media. Treated cells were subjected to 200 Hz for 3 days. After treatment, cells were harvested using trypsin and resuspended in ice-cold FACS buffer and then stained with PI for at least 10 minutes before flow cytometry analysis (BD Fortessa).

### Intracranial xenograft mouse models

The animal study protocol was approved by the Institutional Animal Care and Use Committee (IACUC; AUP-23-024) of the University of California, Irvine. Six- to 8-week-old NOD-SCID gamma (NSG; RRID: BCBC_4142) mice (male and female) were anesthetized using 1.5% to 3% isoflurane. Mice were secured on a stereotaxic device under anesthesia during the procedure. Using a scalpel, an incision was made to reveal sutures on the skull. A burr hole was made using a Dremel at the coordinates −2 mm M/L and +1 A/P from the bregma on the left frontal lobe. A total of 1 × 10^3^ G12-Luc or HOG cells were transplanted at −2.5 mm below the dura using a blunt 30-gauge Hamilton syringe at a controlled rate of 1,000 nL/minute in 2 μL volume. The hole was sealed with bone wax, and the incision site was closed using wound clips. Animals were given postoperative care using pain reliever (Carprofen) and antibiotics with enrofloxacin (Baytril) in accordance with our IACUC-approved protocols. A week following transplantation, mice were given 10 mg/kg (G12-Luc) or 100 mg/kg (HOG) of TMZ or vehicle (10% DMSO in PBS) of equal volume for five consecutive days by intraperitoneal injection. BT9 (30 mg/kg) or compatible vehicle control (200 mg/mL Captisol) was administered by oral gavage once a day for eight consecutive days for the BT9 and TMZ *in vivo* study. Survival was calculated from the day cells were implanted to the day animals were euthanized as established by human endpoint criteria under IACUC guidelines (maximal weight loss >20%, emaciation, impaired mobility, matted fur, hunched posture, and neurologic changes).

### Bioluminescence *in vivo* imaging

Mice were given a single dose of 150 mg/kg of Luciferin (Gold Bio) intraperitoneally and anesthetized with isoflurane for 5 minutes. Mice were then imaged using AMI-HT imager (Spectral Instruments Imaging) and analyzed for bioluminescence (photons/s) to track tumor growth.

### Bioinformatics

*PAM16* was queried on GlioVis (RRID: SCR_025710) using GBM patient data sets from The Cancer Genome Atlas (TCGA) and the Chinese Glioma Genome Atlas (CGGA) to compare IDH wild-type primary and recurrent GBM samples (26). Correlative analyses were performed on primary patient data using Pearson’s correlation on GraphPad.

### Statistical analyses

All experimental data statistics were analyzed using Graph Pad Prism 10 software. qPCR data bar graphs are represented as the mean ± SD. For comparisons of two samples on qPCR data, a standard two-tailed unpaired *t* test was performed with *n* = 4 technical replicates. For three or more qPCR experimental groups, a one-way analysis of variance along with *post hoc* Tukey test was used to compare the means of multiple groups. *P* values are designated as *P < 0.05. Median fluorescence intensity was used to display flow cytometry data and perform statistical analyses. Each independent experiment was repeated with at least three technical replicates. *P* values for survival analyses were calculated using a log-rank test.

## Results

### MAGMAS levels are increased in recurrent GBM patient tumor specimens and TMZ-resistant glioma cell lines

**Figure 1.**
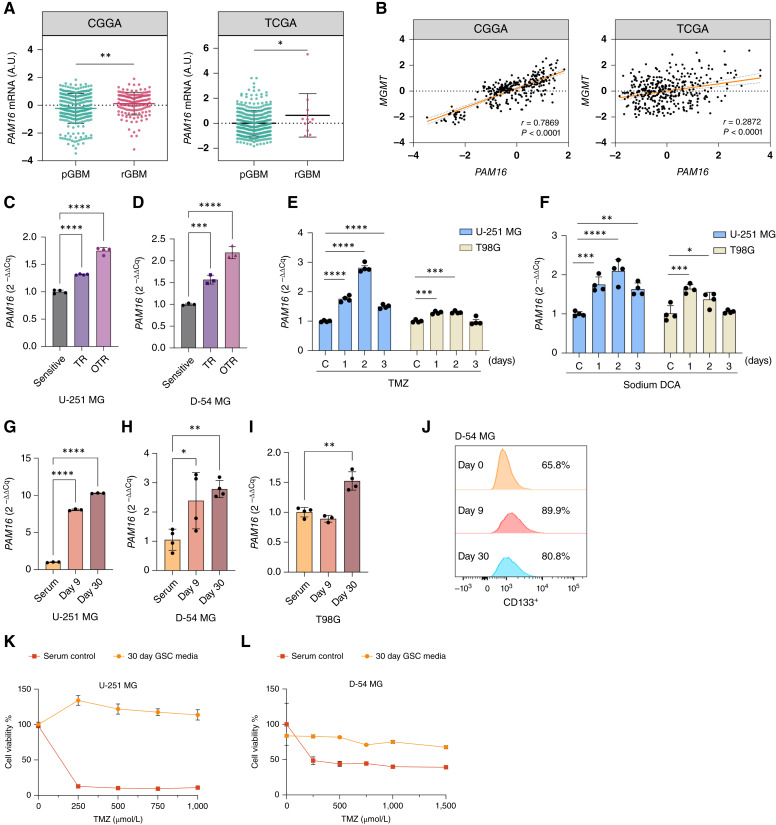
MAGMAS is differentially expressed in newly diagnosed vs. recurrent GBM patient-derived tumor specimens and TMZ-resistant glioma lines. **A,***PAM16* mRNA expression in IDH1 wild-type newly diagnosed and recurrent GBM tumor samples from the CGGA and TCGA datasets. Two-tailed unpaired *t* test statistical analyses was performed. **B,** Correlation between *PAM16* and *MGMT* mRNA expression in primary GBM patient tumor samples. Pearson correlation, CGGA, R = 0.7869, *P* < 0.0001; TCGA, R = 0.2773, *P* < 0.0001. qPCR analysis of *PAM16* in (**C**) U-251 MG S, TR, and OTR and (**D**) D-54 MG S, TR, and OTR lines. U-251 MG S and T98 cells treated with (**E**) 1,000 μmol/L TMZ, (**F**) 30 mmol/L DCA (U-251 MG S), or 34 mmol/L DCA (T98G). RNA was collected at 0 days (vehicle control) and 1-, 2-, and 3-day time points, and qPCR analysis of *PAM16* was performed. One-way ANOVA, *post hoc* Tukey, *P* < 0.05. qPCR analysis of *PAM16* expression in (**G**) U-251 MG S, (**H**) D-54 MG S, and (**I**) T98G cells cultured in GSC media. Cells were collected at 9 and 30 days, and *PAM16* expression was compared with cells grown in serum control media. One-way ANOVA, *post hoc* Tukey, multiple comparisons, *P* < 0.05. **J,** Flow cytometry analysis of CD133 expression of D-54 MG cells at 0, 9, and 30 days cultured in GSC media for dedifferentiation. On 30 days of dedifferentiation, (**K**) U-251 MG and (**L**) D-54 MG were collected and treated with graded doses of TMZ for 5 days; cell viability was assessed by MTT. All data are shown as the mean ± SD; *, *P* < 0.05; **, *P* < 0.01; ***, *P* < 0.001; ****, *P* < 0.0001.

MAGMAS is overexpressed in GBM tumor specimens compared with human normal brain tissue (11). To examine whether MAGMAS levels are overexpressed in recurrent GBM compared with newly diagnosed GBM tumors, we compared the expression levels of *PAM16* (the MAGMAS protein-coding gene) in GBM patient samples using the CGGA and TCGA data sets (Fig. 1A). *PAM16* levels were significantly higher in recurrent tumors of patients who had relapsed compared with tumor specimens of newly diagnosed patients that had not received prior GBM treatment. We also found a strong positive correlation between *PAM16* and *MGMT* expression levels in primary GBM tumor specimens (Fig. 1B). Based on these findings, we hypothesized that MAGMAS overexpression may be associated with TMZ resistance.

We next examined whether MAGMAS levels correlated with TMZ resistance in malignant glioma cell lines using previously established TMZ-resistant cell lines that were either resistant to TMZ alone (U-251 MG TR and D-54 MG TR) or TMZ + O^6^-benzylguanine (U-251 MG OTR and D-54 MG OTR; Supplementary Fig. S1A and S1B; refs. 27, 28). We quantified *PAM16* levels in U-251 MG and D-54 MG cells, as well as in their respective TR and OTR lines. We found that *PAM16* expression levels were significantly increased in all TMZ-resistant lines compared with the TMZ-sensitive parental lines (Fig. 1C and D). *MGMT* mRNA expression levels were also tested, and we observed the highest expression levels in TR and T98G cells but were not detected in D-54 MG parent cells (Supplementary Fig. S1C). O^6^-benzylguanine binds to MGMT, inhibiting MGMT-mediated DNA repair; therefore, inhibiting TMZ-resistant cells dependent on increased MGMT expression (27, 29). *PAM16* expression was higher in the OTR than in the TR-resistant lines, suggesting that MGMT does not directly regulate MAGMAS levels.

Previous studies have reported that MAGMAS levels are higher in normal human and murine tissues with high metabolic demand and high oxygen consumption, including the CNS and cardiac and skeletal muscles (Human Protein Atlas available from www.proteinatlas.org; refs. 30, 31). Additionally, during the acquisition of TMZ resistance in glioma lines, long-term treatment with TMZ results in a significant increase in oxygen consumption starting during the transient resistance phase before reaching a resistant phenotype with reprogrammed mitochondria (32, 33). As MAGMAS controls protein trafficking in the mitochondrial matrix, we hypothesized that MAGMAS levels would increase following exposure to TMZ. We observed higher *PAM16* levels in U-251 MG and T98G cells treated with 1,000 μmol/L TMZ for 3 days compared with vehicle-treated controls (Fig. 1E). To determine whether MAGMAS plays a role in the metabolic switching, going from glycolysis to oxidative phosphorylation (OXPHOS) for adenosine triphosphate (ATP) production, we treated U-251 MG and T98G cells with sodium DCA, a pyruvate dehydrogenase kinase inhibitor, for 3 days to reduce glycolysis by blocking the production of lactic acid (Supplementary Fig. S1D; ref. 34). DCA treatment significantly increased *PAM16* mRNA expression (Fig. 1F) and MAGMAS protein levels (Supplementary Fig. S1E).

GSCs contribute to GBM tumor heterogeneity and are associated with TMZ resistance, which makes them a major contributor to tumor recurrence (35–37). To assess whether MAGMAS levels are higher in GSCs, we dedifferentiated U-251 MG (Fig. 1G), D-54 MG (Fig. 1H), and T98G (Fig. 1I) by culturing them in stem cell serum-free media (GSC media) containing growth factors (bFGF and EGF) and B27, which promote a stem cell–like phenotype, adapted from our previously published methods (21, 38). We observed elevated *PAM16* expression at day 30 in the cell lines cultured in GSC media compared with the control cells maintained in the serum-containing media (Fig. 1G–I). We evaluated the GSC populations throughout the dedifferentiation process by flow cytometry analysis of CD133^+^ cells. There were significantly higher CD133^+^ populations in the GSC media–cultured D-54 MG at days 9 and 30 as compared with the D-54 MG cells grown in control media (Fig. 1J). On day 30, we assessed the viability of dedifferentiated U-251 MG (Fig. 1K) and D-54 MG (Fig. 1L) in response to TMZ and observed increased resistance to TMZ in the dedifferentiated lines cultured in GSC media compared with the control (Fig. 1K and L).

### Pharmacologic inhibition of MAGMAS decreases protein trafficking into the mitochondria

**Figure 2.**
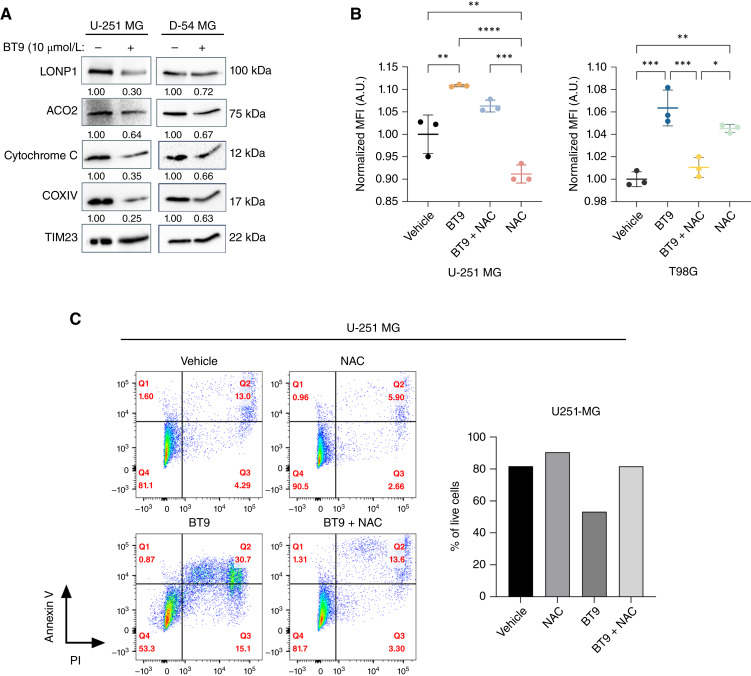
Pharmacologic MAGMAS inhibition by BT9 reduces protein trafficking into the mitochondria and increases oxidative stress in glioma lines U-251 MG, T98G, and D-54 MG. **A,** U-251 MG and D-54 MG were treated with 10 μmol/L BT9 for 24 hours. Mitochondrial lysates were collected, and protein levels of LONP1, ACO2, cytochrome C, COXIV, and TIM23 were assessed by Western blot. TIM23 was used as a loading control. **B,** U-251 MG and T98G cells were treated with 5 μmol/L BT9 and 2.5 mmol/L of NAC for 24 hours and probed with MitoSOX Red. The median fluorescence intensity (MFI) was determined by flow cytometry (*n* = 3 technical replicates). **C,** U-251 MG cells were exposed to 5.6 μmol/L BT9 ± 2.5 mmol/L NAC for 3 days. Cells were then stained with Annexin V/PI and analyzed by flow cytometry to assess early and late apoptosis. Early and late apoptosis events observed for the vehicle were 13%, 5.9% for the NAC control, 30.7% for the BT9-treated cells, and 13.6% for the BT9 and NAC-treated cells. Data representative of one experiment. Data are shown as the mean ± SD; *, *P* < 0.05; **, *P* < 0.01; ***, *P* < 0.001; ****, *P* < 0.0001.

The mitochondrial protein-trafficking pathway is crucial for maintaining mitochondrial homeostasis. Our previous studies demonstrated that the MAGMAS small-molecule inhibitor BT9 was cytotoxic to glioma cells and significantly reduced mitochondrial respiratory function (11). Therefore, we sought to examine whether MAGMAS inhibition with BT9 impairs the trafficking of nuclear-encoded proteins into mitochondria. We treated U-251 MG, D-54 MG, and G12 with a high dose of BT9 (10 μmol/L) for 24 hours. LONP1, COXIV, cytochrome C, and ACO2 protein levels were reduced in the mitochondrial lysates from BT9 treated cells compared with vehicle-treated controls (Fig. 2A; Supplementary Fig. S1F). To expand coverage and determine which mitochondrial proteins are mostly affected, we used SILAC on D-54 MG cells and treated them with a 3 μmol/L dose of BT9 for 24 hours to detect changes in mitochondrial proteins. A gene ontology analysis revealed that significantly reduced proteins were involved in aerobic respiration, tricarboxylic acid cycle, and cellular amino acid catabolic processes (Supplementary Fig. S1G–S1I). Interestingly, we found that SOD2, a mitochondrial protein that binds to superoxide radicals from OXPHOS byproducts and converts them to hydrogen peroxide and diatomic oxygen, protein levels were reduced (Supplementary Fig. S1G and S1H). Based on these results, we hypothesized that BT9 treatment increases superoxide levels in GBM cells. We determined that BT9 significantly increased ROS production, which was partially quenched by the concomitant addition of the antioxidant NAC and BT9 in U-251 MG and T98G cells at 24 hours (Fig. 2B). Next, we tested whether BT9 induces apoptosis and whether NAC can prevent it. BT9 increased the early/late apoptotic (Annexin V^+^/PI^+^) population by 30.7% and the necrotic (Annexin V^−^/PI^+^) population by 15.1% (Fig. 2C). When NAC was added in combination with BT9, the early/late apoptotic population was reduced to 13.6%, and the Annexin V^−^/PI+ cell population to 3.3%, levels which were comparable with the vehicle control and NAC groups, 4.29% and 2.66%, respectively (Fig. 2C).

We also confirmed our data obtained in established malignant glioma lines using a primary glioma stem cell line, G12. BT9 treatment alone (4 μmol/L) reduced the percentage of live G12 cells to 25.9%, TMZ (500 μmol/L) to 38.1%; when used in combination, there was only 11.5% of live G12 cells after 3 days of treatment (Supplementary Fig. S1J). With the addition of 2.5 mmol/L of NAC to BT9- or TMZ-treated cells, cell viability was slightly higher for BT9 plus NAC (28.3% vs. 25.9%) and remained unchanged for TMZ- and NAC-treated cells. However, when combining NAC with BT9 and TMZ, the number of live cells increased to 30.4%. These findings suggest that impaired mitochondrial protein trafficking and increased ROS production are associated with BT9-dependent glioma apoptotic cell death.

### BT9 is effective against TMZ-resistant glioma lines and GSC and has limited toxicity against HAs

**Figure 3.**
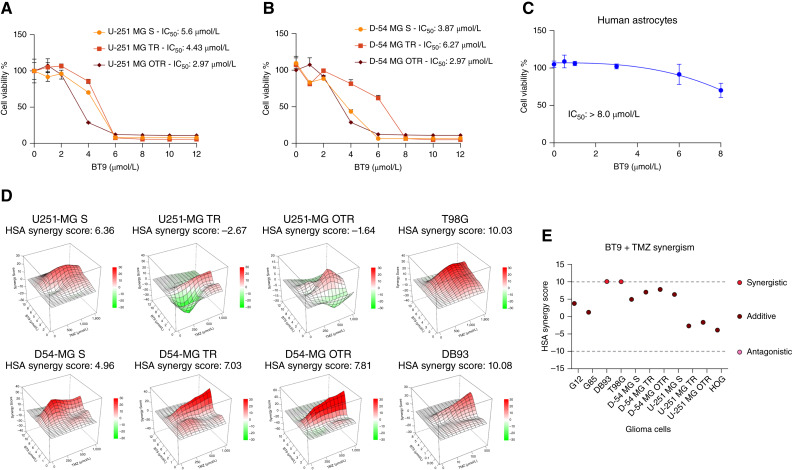
BT9 is effective against TMZ-resistant glioma lines and GSCs, shows synergism with TMZ, and has limited toxicity against HAs. **A,** U-251 MG, (**B**) D-54 MG, and (**C**) normal HAs were treated with BT9 alone for 72 hours, and cell viability was assessed by MTT or XTT assays. **D,** Panel of 3D synergy plots of U-251 MG, D-54 MG, T98G, resistant cells, and GSCs treated with increasing doses of BT9 ± TMZ for 72 hours. **E,** Summary graph of HSA synergy scores of all tested glioma cell lines. Scores were calculated using SynFinder+.

We next evaluated the cytotoxic effect of BT9 in TMZ-sensitive (U-251 MG and D-54 MG) and TMZ-resistant glioma lines (U-251 MG TR, U-251 MG OTR, D-54 MG TR, and D-54 MG OTR). We treated U-251 MG (S, TR, and OTR, Fig. 3A) and D-54 MG (S, TR, and OTR, Fig. 3B) lines with graded doses of BT9 for 3 days and assessed viability using the MTT assay. BT9 exerted a similar reduction in viability in the TMZ-resistant lines (TR and OTR) compared with the parental TMZ-sensitive lines (S), indicating that BT9 can target cells irrespective of their TMZ resistance status (Fig. 3A and B; Table 1). We also examined the cytotoxic effect of BT9 on the patient-derived GSC line DB93 and the Mayo Clinic (39) brain tumor PDX lines (G12 and G85) and determined that they were also similarly sensitive to BT9 (IC_50_: 1.69, 2.02, 3.25 μmol/L, respectively, Table 1). The IC_50_ value of BT9 in normal HAs (IC_50_ >8 μmol/L, Fig. 3C) was higher than that of TMZ-resistant glioma lines and patient-derived GSC and PDX lines, suggesting a good therapeutic index.

**Table 1.** IC_50_ values and HSA scores for BT9 and TMZ.

Cell line	BT9 (μmol/L)	TMZ (μmol/L)	HSA synergy score
U-251 MG (S)	5.6^a^	107.4^b^	6.36^a^
U-251 MG-TR	4.43^a^	>1,000^b^	−2.67^a^
U-251 MG-OTR	2.97^a^	>1,000^b^	−1.64^a^
D-54 MG (S)	3.87^a^	677.1^b^	4.96^a^
D-54 MG-TR	6.27^a^	>1,000^b^	7.03^a^
D-54 MG-OTR	2.97^a^	>1,000^b^	7.81^a^
T98G	4.30^a^	945.4^c^	10.03^a^
G12	2.02^a^	94.15^b^	3.79^a^
G85	3.25^a^	497.5^b^	1.27^a^
DB93	1.69^a^	301.7^c^	10.08^a^
HOG	1.77^a^	17.95^b^	−3.855^a^
HAs	>8^a^	Not tested	Not tested

TMZ treatment time: a = 3 day, b = 5 day, c = 7 day.

### MAGMAS inhibition with BT9 sensitizes GSC and TMZ-resistant glioma cells to TMZ

We next examined whether BT9 could sensitize TMZ-resistant glioma lines and GSC to TMZ when used in combination *in vitro*. Glioma cells were treated with both BT9 (0–10 μmol/L) and TMZ (0–1,000 μmol/L) for 3 days, and cell viability was assessed using the MTT or XTT assays (Fig. 3D). Combined treatment with TMZ and BT9 markedly decreased the viability of the glioma and GSC lines, with the effects greater than those observed in cell lines treated with either drug alone. We used the SynFinder+ web application (https://synergyfinder.org) to calculate the synergy scores for BT9 and TMZ using the highest single agent (HSA) model mathematical model. Combinatory drugs that score below −10 are considered antagonistic, scores between −10 and +10 are additive, and scores above 10 are considered synergistic. Cell lines DB93 and T98G had the highest HSA scores compared with the other cell lines, suggesting that TMZ and BT9 exerted a synergistic reduction in viability (Fig. 3E). All other cell lines had scores between −10 and +10, indicating that the combinatory BT9 + TMZ treatment had an additive cytotoxic effect against TMZ-resistant glioma lines and GSC.

To determine the impact of BT9 or/and in combination with TMZ, we conducted a treatment study using luciferase expressing G12 cells (G12-Luc) to transplant into immunodeficient mice (NOD-SCID) as our mouse GBM model. We transplanted 1 × 10^3^ G12-Luc cells into the left frontal lobe and allowed them to expand. After 7 days, mice were treated with 30 mg/kg of BT9 by oral gavage for eight consecutive days or 10 mg/kg of TMZ (intraperitoneally.) for five consecutive days (Supplementary Fig. S2A). No significant differences were observed between vehicle- or BT9-treated mice (*P* = 0.5005; Supplementary Fig. S2B). However, TMZ treatment alone resulted in significantly higher OS when compared with vehicle and BT9 groups (*P* = 0.0015 and *P* = 0.0018, respectively). When BT9 was used in combination with TMZ, we observed an increase in survival when compared with TMZ alone (*P* = 0.0790) with one mouse showing no signs of health decline or tumor growth (Supplementary Fig. S2C–S2E). OS of BT9- and TMZ-treated mice when compared with vehicle and BT9 alone was significantly higher (*P* = 0.0038 and *P* = 0.0049, respectively).

### MAGMAS knockdown in GBM cells increases sensitivity to TMZ, radiation, and TTFs

**Figure 4.**
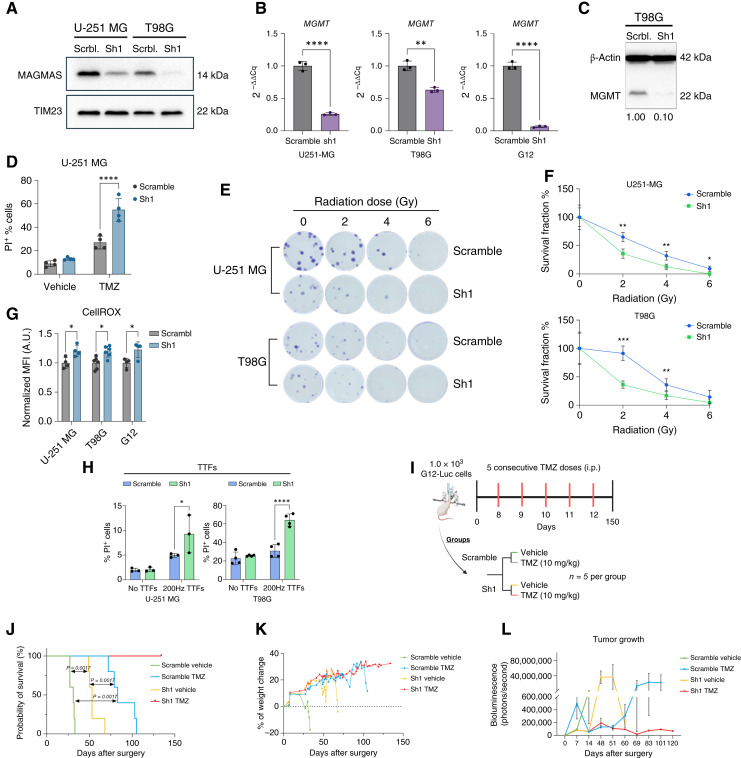
MAGMAS knockdown in GBM cells increases sensitivity to TMZ, radiation, and TTFs. **A,** Western blot analysis of MAGMAS protein levels in mitochondrial lysates from U-251 MG and T98G cells lentiviral transduced with either scramble control or sh*PAM16*. TIM23 was used as a loading control for each sample. **B,***MGMT* mRNA expression was quantified by qPCR in U-251 MG, T98G, and G12 scramble and sh*PAM16* cell lines. **C,** Whole cell lysates harvested from T98G scramble and sh1 cells probed with anti-MGMT (bottom bands) and Β-actin (top bands) as a loading control. Membrane is a duplicate of Supplementary Fig. S3D. Only T98G cells had detectable MGMT at the protein level which is highlighted here. **D,***PAM16* knockdown U-251 MG cells were treated with 24 μmol/L TMZ for 5 days, and the percent of PI+ cells were determined by flow cytometry (*n* = 4). **E,** Cellular ROS measured from U-251 MG, T98G, and G12 *PAM16* knockdown cells with stained with CellROX and analyzed by flow cytometry (*n* = 4). Statistical analyses were done with unpaired *t* test, mean ± SD. **F,** Representative images of clonogenic assay plates and (**G**) quantification of surviving fraction of U-251 MG and T98G scramble control and *PAM16* knockdown cells exposed to 0 to 6 Gy of radiation. Colonies were counted manually. Statistical analyses were performed using the mixed-effects model, Sidak multiple comparisons test, *P* < 0.05, mean with ± SD. MFI, median fluorescence intensity. **H,** U-251 MG and T98G scramble control and *PAM16* knockdown cells were exposed to 200 Hz TTFs for 3 days. Cell viability was assessed by quantifying the percent of PI+ cells using flow cytometry. Significance was determined using a two-way ANOVA, Sidak multiple comparisons test, *P* < 0.05. **I,** Timeline and experimental design of the G12-Luc orthotopic xenograft model. Scramble + vehicle: *n* = 5; Scramble + TMZ; *n* = 5; Sh1 + vehicle: *n* = 5; Sh1 + TMZ: *n* = 5. **J,** Kaplan–Meier curve depicting survival of *PAM16* knockdown transduced G12-Luc orthotopic xenograft mouse model. **K,** Summary of bioluminescence imaging of mice from all treatment groups (photons/s) from different time points of the study. **L,** The change in body weights of mice was measured at different time points throughout the study. Log-rank *P* values, Scrbl. vehicle vs. Sh1 vehicle *P* = 0.0017, Scrbl. vehicle vs. Scrbl. TMZ *P* = 0.0017, Scrbl. vehicle vs. Sh1 TMZ *P* = 0.0017, Scrbl. TMZ vs. Sh1 vehicle *P* = 0.0021, Scrbl. TMZ vs. Sh1 TMZ *P*= 0.0018, and Sh1 vehicle vs. Sh1 TMZ *P* = 0.0021. Data are shown as the mean ± SD; *, *P* < 0.05; **, *P* < 0.01; ***, *P* < 0.001; ****, *P* < 0.0001. [**I,** Created in BioRender. Lepe, J. (2026) https://BioRender.com/wvy3h4p].

To validate our pharmacologic findings with the MAGMAS inhibitor BT9 (Supplementary Fig. S3A), we genetically manipulated MAGMAS expression in U-251 MG, T98G, G12, and HOG cells and generated *PAM16* knockdown lines. Using lentiviral particles packaged with *PAM16* shRNA (Sh1) or scramble control, we transduced U-251 MG, T98G, G12, and HOG cells and extracted mitochondrial lysates and verified knockdown by Western blot (Fig. 4A; Supplementary Fig. S3B). *MGMT* expression was significantly lower in *PAM16* knockdown U-251 MG, T98G, G12, and HOG (Fig. 4B and C; Supplementary Fig. S3C) cells when compared with the scramble controls. Additionally, we probed whole cell lysates from U-251 MG, T98G, and G12 knockdown cells with NRF1 antibody, a transcription factor that is known to regulate MGMT, and found that NRF1 protein levels were reduced in knockdown cells (Supplementary Fig. S3D). *PAM16* knockdown sensitized U-251 MG and HOG to TMZ-induced cell death (Fig. 4D; Supplementary Fig. S3E). *PAM16* knockdown alone did not decrease the viability of U-251 MG compared with the scramble control. In addition, we also investigated whether *PAM16* knockdown changed cellular ROS levels. We found a significant increase in ROS in U-251 MG, T98G, and G12 knockdown cells (Fig. 4E). Using a colony formation assay, *PAM16* knockdown in T98G and U-251 MG cells resulted in reduced clonogenic capacity compared with the scramble control cell lines (Supplementary Fig. S3F and S3G).

We next examined the impact of MAGMAS silencing on sensitivity to radiotherapy. The shRNA control and *PAM16* knockdown cells were exposed to a range of radiation doses (0–6 Gy), and colony formation was examined 8 days after irradiation. Exposure to increasing doses of radiation resulted in a dose-dependent reduction in colony formation in *PAM16*-knockdown cells, with the most significant decrease observed in the 2 and 6 Gy irradiated MAGMAS-deficient U-251 MG and T98G cells (Fig. 4F and G). Next, we assessed whether *PAM16* knockdown sensitized U-251 MG and T98G cells to TTFs using the Novocure Inovitro system (40). The cells were exposed to an electrical frequency of 200 Hz for 3 days and then assessed for viability using PI staining by flow cytometry (Fig. 4H). TTFs increased the percentage of PI+ cells in scramble and *PAM16* knockdown cells in both cell lines compared with the respective no-TTF controls. *PAM16* knockdown in U-251 MG and T98G cells sensitized them to TTF-induced cell death when compared with their scramble controls (Fig. 4H).

### MAGMAS knockdown in combination with TMZ increases OS in an orthotopic glioma mouse model

To determine whether *PAM16* knockdown sensitized tumors to TMZ *in vivo*, using a primary GBM patient cell line, G12-Luc-Scramble and G12-Luc-Sh1, we transplanted intracranially into immunodeficient NSG mice. A total of 1 × 10^3^ cells were transplanted into the left frontal lobe of each mouse and allowed to expand for 8 days before treatment (Fig. 4I). After randomization (*n* = 5 per group), the mice were treated with either vehicle or 10 mg/kg of TMZ for five consecutive days starting on day 8. Scramble TMZ mice had significantly longer OS than the scramble vehicle group (*P* = 0.0017), with median survival of 31 and 83 days, respectively (Fig. 4J). The Sh1 vehicle group also had a significantly longer median survival time than the scramble vehicle group (*P* = 0.0017) at 53 days. Remarkably, Sh1-TMZ–treated mice survived throughout the entirety of the study with no signs of health decline or detectable tumor at the end of the study (vs. scramble vehicle *P* = 0.0017, vs. scramble TMZ *P* = 0.0018, and vs. sh1 vehicle *P* = 0.0021, respectively (Fig. 4J–L).

Next, we confirmed the *in vivo* effect of *PAM16* knockdown on an orthotopic anaplastic astrocytoma model in NSG mice using human malignant glioma (HOG) tumor cells selected in stem-cell media (21). A total of 1 × 10^3^ cells of either scramble or Sh1 HOG cells were transplanted into the left frontal lobe of mice (Supplementary Fig. S3H). Seven days after transplantation, vehicle or 100 mg/kg TMZ was administered intraperitoneally daily for five consecutive days. The animals were observed for 150 days, and OS was assessed in all experimental groups (Supplementary Fig. S3I). Mice transplanted with Sh1 HOG cells that received TMZ survived significantly longer (median OS >150 days) compared with mice in the Sh1 group that received vehicle and scramble TMZ treatment (*P* = 0.0002 and *P* = 0.0597, respectively). There was no difference in survival between the scramble and Sh1 groups that received vehicle; both had the same median survival of 22 days (*P* = 0.358). After TMZ treatment, mice bearing Sh1 tumors had a higher body weight compared with mice in the scramble TMZ-treated group (Supplementary Fig. S3J). These findings suggest that *PAM16* knockdown can effectively be combined with TMZ to prolong survival in orthotopic xenograft glioma mouse models.

## Discussion

GBM is one of the most aggressive human CNS cancers that affects three in 100,000 adults (1, 41, 42). Despite patients receiving the best standard of care, median survival is only 14.6 months, OS for 5 years is 50%, and only 1% of patients survive up to 10 years (2). The current standard of care includes maximal surgical resection, radiation, and chemotherapy with TMZ. In addition, TTFs have been FDA-approved for newly diagnosed and recurrent patients with GBM. However, current treatments only modestly prolong the life of patients, and most tumors recur within the first year of the initial diagnosis (43, 44).

Factors that contribute to GBM mechanisms of resistance are multiple and include tumor heterogeneity, the immunosuppressive tumor microenvironment, GSCs, mitochondrial reprogramming, and the activation of DNA repair pathways (35–37, 45–47). In the present study, we identified that the mitochondrial protein MAGMAS is overexpressed in recurrent GBM tumors compared with newly diagnosed GBM samples, TMZ-resistant glioma lines, and GSCs. We also found that *PAM16* expression positively correlated with *MGMT*, suggesting that *PAM16* may be involved in mitochondrial-mediated TMZ resistance mechanisms. MAGMAS regulates protein trafficking in the mitochondrial matrix compartment by regulating the ATPase stimulatory activity of DNAJC19 and functions as an ROS regulator (18, 48–51). Cancer cells are known to favor aerobic glycolysis through the breakdown of glucose for intermediate byproducts to generate ATP and biomass for proliferation (52). However, resistant cells that survive TMZ exposure undergo metabolic alteration by switching from aerobic glycolysis to OXPHOS to generate ATP (53, 54). MAGMAS overexpression may facilitate mitochondrial protein-trafficking and has been shown to reduce ROS production and enhance ETC activity, which may promote the metabolic alterations observed in GBM (18).

The cell surface marker CD133 (*PROM1*) is a widely used marker to identify cancer stem cells across various cancers, including GBM (36, 46). Studies have shown that CD133 regulates several essential pathways related to chemotherapy resistance, quiescence, tumor recurrence, heterogeneity, and cell differentiation (46, 54). In this study, we observed significantly higher *PAM16* and CD133 expression, including during the acquisition of TMZ resistance in established glioma cell lines, that were dedifferentiated into a more GSC-like phenotype using serum-free media conditions that promote stem cell enrichment.

Glioma cells exposed to sublethal doses of TMZ enhance their mitochondrial function by increasing oxygen consumption during a phenotypic transitory phase before reaching a TMZ-resistant state (32). In this study, cells treated with TMZ over 3 days showed a significant increase in *PAM16* expression. Additionally, cells treated with DCA, a pyruvate dehydrogenase kinase inhibitor, also significantly increased *PAM16*, suggesting that increased MAGMAS levels help facilitate metabolic reprogramming by increasing the protein-trafficking capacity to help bring in proteins into the mitochondria matrix and relieve mitochondrial dysfunction (21, 55, 56).

**Figure 5.**
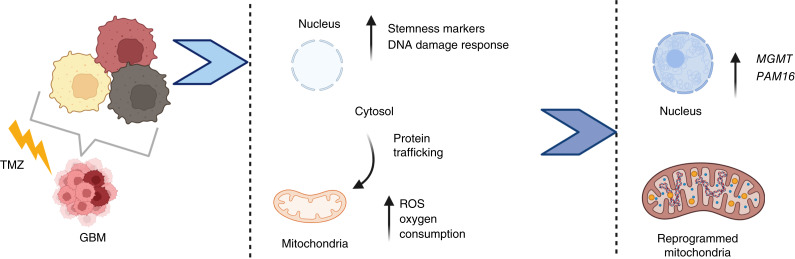
Schematic illustration of chemotherapy resistance acquisition in glioma cells. Glioma cells treated with TMZ activate early response mechanisms that include increased stemness marker (*PROM1*), OXPHOS activity, and increased MAGMAS expression to facilitate protein trafficking into mitochondria. DNA damage response mechanisms are also activated to help repair alkylated DNA. In the later stages of TMZ resistance acquisition, MGMT levels are elevated to remove the alkylated O6-methylguanine from DNA, making glioma cells more resistant to TMZ. MAGMAS levels are sustained at higher levels in chemotherapy-resistant cells with reprogrammed mitochondria, providing increased protein-trafficking capacity in mitochondria, which requires higher oxygen consumption and mitigates ROS toxicity. [Created in BioRender. Lepe, J. (2026) https://BioRender.com/ilvryag].

Our laboratory and others previously reported that pharmacologic inhibition of MAGMAS with BT9 is cytotoxic to cancer cells, decreases oxygen consumption, and induces apoptosis through cleaved caspase-3 activation (11, 13, 20, 55, 56). We expanded upon these findings in this study and found that TMZ-resistant glioma lines exhibit a similar response to BT9 as the TMZ-sensitive parental lines (IC_50_ = 2.9–6.3 μmol/L). Based on these findings, we hypothesized that BT9 reduces oxygen consumption by blocking the shuttling of precursor protein subunits needed to assemble the ETC complexes and additional proteins involved in mitochondrial homeostasis. Our biochemical data demonstrate that BT9 reduced mitochondrial-targeted proteins required for OXPHOS machinery and increased superoxide levels, contributing to ROS production. Given that in the early stages of resistance acquisition, upon acute TMZ exposure, GBM cells upregulate oxygen consumption, we combined BT9 with TMZ and found that the combinatorial treatment performed synergistically in all tested glioma cell lines (32, 53). Additionally, combinatorial treatment of BT9 with TMZ *in vivo* resulted in an increase in OS over TMZ treatment alone. These findings demonstrate that BT9 enhances the efficacy of TMZ in GBM cells, regardless of resistance status, supporting its clinical potential for patients with both primary and recurrent tumors (Fig. 5).

When we knocked down MAGMAS levels in U-251 MG, T98G, G12, and HOG cells and treated them with TMZ, radiation, or TTFs, we found that MAGMAS-deficient cells were more sensitive to each of these treatments than scramble controls. Additionally, we transplanted G12-Luc and HOG *PAM16* knockdown cells as an orthotopic glioma model in NSG mice and treated them with vehicle or 10 and 100 mg/kg TMZ, respectively. Although there were no statistical differences in survival between the scramble controls and sh*PAM16* knockdown in HOG cells, we did see significant differences in G12-Luc knockdown cells, suggesting that the GSCs are more sensitive to the MAGMAS disruption. Additionally, mice implanted with sh*PAM16* knockdown cells and treated with TMZ had significantly higher OS than the other groups. These findings suggest that MAGMAS inhibition, combined with already existing treatments, may be of future clinical relevance for GBM.

### Conclusion

Most patients with GBM experience tumor recurrence within a year after treatment and have limited clinical treatment options due to resistance to chemotherapy and radiation. To develop novel therapeutic compounds that can be used in combination with the standard of care, it is necessary to understand which cellular response mechanisms are triggered at the acute and late stages of resistance after cells are exposed to chemotherapy. Our findings suggest that MAGMAS plays an important role in chemotherapy resistance and its suppression sensitizes resistant cells to TMZ through concomitant treatment. By inhibiting MAGMAS, we suppress trafficking of essential nuclear-encoded proteins, including LONP1, ACO2, COXIV, and cytochrome C, into the mitochondria. These critical proteins help maintain mitochondrial homeostasis and OXPHOS energy metabolism. LONP1 is an essential mitochondrial protein that is highly expressed in different cancers and correlates with poor prognosis (21). Reducing levels of LONP1 further increases mitochondrial stress, ROS accumulation, and impaired bioenergetics, as previously reported (57). Additionally, inhibition of this pathway increases proteolytic stress and reduces energy metabolism, which makes the cells vulnerable to TMZ. Further investigation is needed to elucidate the MAGMAS mechanism of action. In summary, our findings suggest that disrupting mitochondrial homeostasis through the inhibition of MAGMAS, and consequently mitochondrial protein trafficking, may restore sensitivity to TMZ in TMZ-resistant GBM thereby, providing a potential therapeutic strategy to improve outcomes in patients with GBM.

## Supplementary Material

Supplementary Figure S1 Figure S1. TMZ resistance in GBM cells and proteomic changes after MAGMAS inhibition.

Supplementary Figure S2 Figure S2. Combinatorial BT9 + TMZ treatment of human primary cell line in orthotopic mouse model.

Supplementary Figure S3 Figure S3. Testing of human glioma cells for TMZ sensitivity.

Supplementary Table S1 Table S1. Cell Line MGMT Methylation Status

Supplementary Table S2 Table S2. qPCR Primer List

## Data Availability

The preprocessed RNA sequencing datasets used for this study were downloaded from GlioVis (RRID: SCR_025710) data portal. All other data used in this study are available upon request from the corresponding author.

## References

[1] Wu W , Klockow JL, Zhang M, Lafortune F, Chang E, Jin L, . Glioblastoma multiforme (GBM): an overview of current therapies and mechanisms of resistance. Pharmacol Res 2021;171:105780. 34302977 10.1016/j.phrs.2021.105780 PMC8384724

[2] Stupp R , Mason WP, van den Bent MJ, Weller M, Fisher B, Taphoorn MJB, . Radiotherapy plus concomitant and adjuvant temozolomide for glioblastoma. N Engl J Med 2005;352:987–96. 15758009 10.1056/NEJMoa043330

[3] Zhang J , Stevens MFG, Bradshaw TD. Temozolomide: mechanisms of action, repair and resistance. Curr Mol Pharmacol 2012;5:102–14. 22122467 10.2174/1874467211205010102

[4] Chen X , Zhang M, Gan H, Wang H, Lee J-H, Fang D, . A novel enhancer regulates MGMT expression and promotes temozolomide resistance in glioblastoma. Nat Commun 2018;9:2949. 30054476 10.1038/s41467-018-05373-4 PMC6063898

[5] Hegi ME , Diserens AC, Gorlia T, Hamou MF, de Tribolet N, Weller M, . MGMT gene silencing and benefit from temozolomide in glioblastoma. N Engl J Med 2005;352:997–1003. 15758010 10.1056/NEJMoa043331

[6] Brandes AA , Franceschi E, Paccapelo A, Tallini G, De Biase D, Ghimenton C, . Role of MGMT methylation status at time of diagnosis and recurrence for patients with glioblastoma: clinical implications. Oncologist 2017;22:432–7. 28275120 10.1634/theoncologist.2016-0254 PMC5388380

[7] Quinn JA , Jiang SX, Reardon DA, Desjardins A, Vredenburgh JJ, Rich JN, . Phase II trial of temozolomide plus o6-benzylguanine in adults with recurrent, temozolomide-resistant malignant glioma. J Clin Oncol 2009;27:1262–7. 19204199 10.1200/JCO.2008.18.8417 PMC2667825

[8] Guo X , Yang X, Wu J, Yang H, Li Y, Li J, . Tumor-treating fields in glioblastomas: past, present, and future. Cancers (Basel) 2022;14:3669. 35954334 10.3390/cancers14153669 PMC9367615

[9] Stupp R , Taillibert S, Kanner A, Read W, Steinberg D, Lhermitte B, . Effect of tumor-treating fields plus maintenance temozolomide vs maintenance temozolomide alone on survival in patients with glioblastoma: a randomized clinical trial. JAMA 2017;318:2306–16. 29260225 10.1001/jama.2017.18718 PMC5820703

[10] Li H-Y , Feng Y-H, Lin C-L, Hsu T-I. Mitochondrial mechanisms in temozolomide resistance: unraveling the complex interplay and therapeutic strategies in glioblastoma. Mitochondrion 2024;75:101836. 38158149 10.1016/j.mito.2023.101836

[11] Di K , Lomeli N, Bota DA, Das BC. Magmas inhibition as a potential treatment strategy in malignant glioma. J Neurooncol 2019;141:267–76. 30414099 10.1007/s11060-018-03040-8 PMC6474352

[12] Jubinsky PT , Short MK, Mutema G, Morris RE, Ciraolo GM, Li M. Magmas expression in neoplastic human prostate. J Mol Histol 2005;36:69–75. 15704001 10.1007/s10735-004-3840-8

[13] Yang J , Das BC, Aljitawi O, Kumar A, Das S, Van Veldhuizen P. Magmas inhibition in prostate cancer: a novel target for treatment-resistant disease. Cancers (Basel) 2022;14:2732. 35681713 10.3390/cancers14112732 PMC9179500

[14] Tagliati F , Gagliano T, Gentilin E, Minoia M, Molè D, Delgi Uberti EC, . Magmas overexpression inhibits staurosporine induced apoptosis in rat pituitary adenoma cell lines. PLoS One 2013;8:e75194. 24069394 10.1371/journal.pone.0075194 PMC3775776

[15] Ahmed N , Kadife E, Raza A, Short M, Jubinsky PT, Kannourakis G. Ovarian cancer, cancer stem cells and current treatment strategies: a potential role of Magmas in the current treatment methods. Cells 2020;9:719. 32183385 10.3390/cells9030719 PMC7140629

[16] Sinha D , Joshi N, Chittoor B, Samji P, D’Silva P. Role of Magmas in protein transport and human mitochondria biogenesis. Hum Mol Genet 2010;19:1248–62. 20053669 10.1093/hmg/ddq002 PMC2838536

[17] Waingankar TP , D’Silva P. Multiple variants of the human presequence translocase motor subunit Magmas govern the mitochondrial import. J Biol Chem 2021;297:101349. 34715125 10.1016/j.jbc.2021.101349 PMC8605242

[18] Srivastava S , Sinha D, Saha PP, Marthala H, D’Silva P. Magmas functions as a ROS regulator and provides cytoprotection against oxidative stress-mediated damages. Cell Death Dis 2014;5:e1394. 25165880 10.1038/cddis.2014.355 PMC4454327

[19] Jubinsky PT , Short MK, Ghanem M, Das BC. Design, synthesis, and biological activity of novel Magmas inhibitors. Bioorg Med Chem Lett 2011;21:3479–82. 21514823 10.1016/j.bmcl.2011.03.050

[20] Motahari Z , Lepe JJ, Bautista MR, Hoerig C, Plant-Fox AS, Das B, . Preclinical assessment of MAGMAS inhibitor as a potential therapy for pediatric medulloblastoma. PLoS One 2024;19:e0300411. 39436961 10.1371/journal.pone.0300411 PMC11495579

[21] Douglas C , Jain S, Lomeli N, Lepe J, Di K, Nandwana NK, . Dual targeting of the mitochondrial Lon peptidase 1 and the chymotrypsin-like proteasome activity as a potential therapeutic strategy in malignant astrocytoma models. Pharmacol Res 2025;215:107697. 40088962 10.1016/j.phrs.2025.107697 PMC12952243

[22] Seidel S , Garvalov BK, Acker T. Isolation and culture of primary glioblastoma cells from human tumor specimens. Methods Mol Biol 2015;1235:263–75. 25388399 10.1007/978-1-4939-1785-3_19

[23] Chiu M , Taurino G, Bianchi M, Ottaviani L, Andreoli R, Ciociola T, . Oligodendroglioma cells lack glutamine synthetase and are auxotrophic for glutamine, but do not depend on glutamine anaplerosis for growth. Int J Mol Sci 2018;19:1099. 29642388 10.3390/ijms19041099 PMC5979401

[24] Louis DN , Perry A, Wesseling P, Brat DJ, Cree IA, Figarella-Branger D, . The 2021 WHO Classification of Tumors of the Central Nervous System: a summary. Neuro Oncol 2021;23:1231–51. 34185076 10.1093/neuonc/noab106 PMC8328013

[25] Wieckowski MR , Giorgi C, Lebiedzinska M, Duszynski J, Pinton P. Isolation of mitochondria-associated membranes and mitochondria from animal tissues and cells. Nat Protoc 2009;4:1582–90. 19816421 10.1038/nprot.2009.151

[26] Bowman RL , Wang Q, Carro A, Verhaak RGW, Squatrito M. Gliovis data portal for visualization and analysis of brain tumor expression datasets. Neuro Oncol 2017;19:139–41. 28031383 10.1093/neuonc/now247 PMC5193031

[27] Bektas M , Johnson S, Poe W, Bigner D, Friedman H. A sphingosine kinase inhibitor induces cell death in temozolomide resistant glioblastoma cells. Cancer Chemother Pharmacol 2009;64:1053–8. 19597728 10.1007/s00280-009-1063-0 PMC3674501

[28] Bota DA , Alexandru D, Keir ST, Bigner D, Vredenburgh J, Friedman HS. Proteasome inhibition with bortezomib induces cell death in GBM stem-like cells and temozolomide-resistant glioma cell lines, but stimulates GBM stem-like cells’ VEGF production and angiogenesis. J Neurosurg 2013;119:1415–23. 24093630 10.3171/2013.7.JNS1323 PMC4550014

[29] Cui B , Johnson SP, Bullock N, Ali-Osman F, Bigner DD, Friedman HS. Decoupling of DNA damage response signaling from DNA damages underlies temozolomide resistance in glioblastoma cells. J Biomed Res 2010;24:424–35. 23554659 10.1016/S1674-8301(10)60057-7 PMC3596690

[30] Jubinsky PT , Short MK, Mutema G, Witte DP. Developmental expression of Magmas in murine tissues and its co-expression with the GM-CSF receptor. J Histochem Cytochem 2003;51:585–96. 12704206 10.1177/002215540305100504

[31] Uhlén M , Fagerberg L, Hallström BM, Lindskog C, Oksvold P, Mardinoglu A, . Tissue-based map of the human proteome. Science 2017;347:1260419. 10.1126/science.1260419 25613900

[32] Rabé M , Dumont S, Álvarez-Arenas A, Janati H, Belmonte-Beitia J, Calvo GF, . Identification of a transient state during the acquisition of temozolomide resistance in glioblastoma. Cell Death Dis 2020;11:19. 31907355 10.1038/s41419-019-2200-2 PMC6944699

[33] Oliva CR , Nozell SE, Diers A, McClugage SG 3rd, Sarkaria JN, Markert JM, . Acquisition of temozolomide chemoresistance in gliomas leads to remodeling of mitochondrial electron transport chain. J Biol Chem 2010;285:39759–67. 20870728 10.1074/jbc.M110.147504 PMC3000957

[34] Tataranni T , Piccoli C. Dichloroacetate (DCA) and cancer: an overview towards clinical applications. Oxid Med Cell Longev 2019;2019:8201079. 31827705 10.1155/2019/8201079 PMC6885244

[35] Bao S , Wu Q, McLendon RE, Hao Y, Shi Q, Hjelmeland AB, . Glioma stem cells promote radioresistance by preferential activation of the DNA damage response. Nature 2006;444:756–60. 17051156 10.1038/nature05236

[36] Liu Q , Nguyen DH, Dong Q, Shitaku P, Chung K, Liu OY, . Molecular properties of CD133^+^ glioblastoma stem cells derived from treatment-refractory recurrent brain tumors. J Neurooncol 2009;94:1–19. 19468690 10.1007/s11060-009-9919-z PMC2705704

[37] Wei Y , Chen Q, Huang S, Liu Y, Li Y, Xing Y, . The interaction between DNMT1 and high-mannose CD133 maintains the slow-cycling state and tumorigenic potential of glioma stem cell. Adv Sci (Weinh) 2022;9:e2202216. 35798319 10.1002/advs.202202216 PMC9475542

[38] Di K , Linskey ME, Bota DA. TRIM11 is overexpressed in high-grade gliomas and promotes proliferation, invasion, migration and glial tumor growth. Oncogene 2013;32:5038–47. 23178488 10.1038/onc.2012.531 PMC3766389

[39] Vaubel RA , Tian S, Remonde D, Schroeder MA, Mladek AC, Kitange GJ, . Genomic and phenotypic characterization of a broad panel of patient-derived xenografts reflects the diversity of glioblastoma. Clin Cancer Res 2020;26:1094–104. 31852831 10.1158/1078-0432.CCR-19-0909 PMC7056576

[40] Kessler AF , Frömbling GE, Gross F, Hahn M, Dzokou W, Ernestus RI, . Effects of tumor treating fields (TTFields) on glioblastoma cells are augmented by mitotic checkpoint inhibition. Cell Death Discov 2018;4:12. 10.1038/s41420-018-0079-9 PMC6125382 30210815

[41] Fares J , Wan Y, Mair R, Price SJ. Molecular diversity in isocitrate dehydrogenase-wild-type glioblastoma. Brain Commun 2024;6:fcae108. 38646145 10.1093/braincomms/fcae108 PMC11032202

[42] Pellerino A , Caccese M, Padovan M, Cerretti G, Lombardi G. Epidemiology, risk factors, and prognostic factors of gliomas. Clin Transl Imaging 2022;10:467–75.

[43] Ballo MT , Conlon P, Lavy-Shahaf G, Kinzel A, Vymazal J, Rulseh AM. Association of Tumor Treating Fields (TTFields) therapy with survival in newly diagnosed glioblastoma: a systematic review and meta-analysis. J Neurooncol 2023;164:1–9. 37493865 10.1007/s11060-023-04348-w PMC10462574

[44] Vaz-Salgado MA , Villamayor M, Albarrán V, Alía V, Sotoca P, Chamorro J, . Recurrent glioblastoma: a review of the treatment options. Cancers (Basel) 2023;15:4279. 37686553 10.3390/cancers15174279 PMC10487236

[45] Perazzoli G , Prados J, Ortiz R, Caba O, Cabeza L, Berdasco M, . Temozolomide resistance in glioblastoma cell lines: implication of MGMT, MMR, P-glycoprotein and CD133 expression. PLoS One 2015;10:e0140131. 26447477 10.1371/journal.pone.0140131 PMC4598115

[46] Abdoli Shadbad M , Nejadi Orang F, Baradaran B. CD133 significance in glioblastoma development: in silico and in vitro study. Eur J Med Res 2024;29:154. 38448914 10.1186/s40001-024-01754-2 PMC10918901

[47] Annovazzi L , Caldera V, Mellai M, Riganti C, Battaglia L, Chirio D, . The DNA damage/repair cascade in glioblastoma cell lines after chemotherapeutic agent treatment. Int J Oncol 2015;46:2299–308. 25892134 10.3892/ijo.2015.2963 PMC4441296

[48] Pais JE , Schilke B, Craig EA. Reevaluation of the role of the Pam18:Pam16 interaction in translocation of proteins by the mitochondrial Hsp70-based import motor. Mol Biol Cell 2011;22:4740–9. 22031295 10.1091/mbc.E11-08-0715 PMC3237618

[49] Sinha D , Srivastava S, D’Silva P. Functional diversity of human mitochondrial J-proteins is independent of their association with the inner membrane presequence translocase. J Biol Chem 2016;291:17345–59. 27330077 10.1074/jbc.M116.738146 PMC5016132

[50] Li Y , Dudek J, Guiard B, Pfanner N, Rehling P, Voos W. The presequence translocase-associated protein import motor of mitochondria. Pam16 functions in an antagonistic manner to Pam18. J Biol Chem 2004;279:38047–54. 15218029 10.1074/jbc.M404319200

[51] D’Silva PR , Schilke B, Walter W, Craig EA. Role of Pam16’s degenerate J domain in protein import across the mitochondrial inner membrane. Proc Natl Acad Sci U S A 2005;102:12419–24. 16105940 10.1073/pnas.0505969102 PMC1194952

[52] Liberti MV , Locasale JW. The Warburg effect: how does it benefit cancer cells? Trends Biochem Sci 2016;41:211–18. 26778478 10.1016/j.tibs.2015.12.001 PMC4783224

[53] Caragher S , Miska J, Shireman J, Park CH, Muroski M, Lesniak MS, . Temozolomide treatment increases fatty acid uptake in glioblastoma stem cells. Cancers (Basel) 2020;12:3126. 33114573 10.3390/cancers12113126 PMC7693784

[54] Oliver L , Lalier L, Salaud C, Heymann D, Cartron PF, Vallette FM. Drug resistance in glioblastoma: are persisters the key to therapy? Cancer Drug Resist 2020;3:287–301. 35582442 10.20517/cdr.2020.29 PMC8992484

[55] Durán AM , Whitley K, Santiago K, Yoo C, Valdez G, Cheng KW, . Inhibition of mitochondrial-associated protein MAGMAS resensitizes chemoresistant prostate cancer cells to docetaxel. Cancers (Basel) 2025;17:1535. 40361461 10.3390/cancers17091535 PMC12072152

[56] Raza A , Hoque A, Luwor R, Escalona RM, Kelly J, Sharma R, . Enhanced expression of mitochondrial Magmas protein in ovarian carcinomas: Magmas inhibition facilitates antitumour effects, signifying a novel approach for ovarian cancer treatment. Cells 2025;14:655. 40358179 10.3390/cells14090655 PMC12071367

[57] Ghosh JC , Seo JH, Agarwal E, Wang Y, Kossenkov AV, Tang HY, . Akt phosphorylation of mitochondrial Lonp1 protease enables oxidative metabolism and advanced tumor traits. Oncogene 2019;38:6926–39. 31406245 10.1038/s41388-019-0939-7 PMC6814529

