# Supplementary material for: MAGMAS Inhibition Enhances Temozolomide Efficacy in Chemotherapy-Resistant Glioblastoma Models
Source: Cancer Res Commun. 2026 Jun 9;6(6):1351–63. doi: 10.1158/2767-9764.CRC-25-0493 (PMC13247981; doi:10.1158/2767-9764.CRC-25-0493)
Supplement: Supplementary Figure S1 — Figure S1. TMZ resistance in GBM cells and proteomic changes after MAGMAS inhibition. [file crc-25-0493_supplementary_figure_s1_suppsf1.docx]

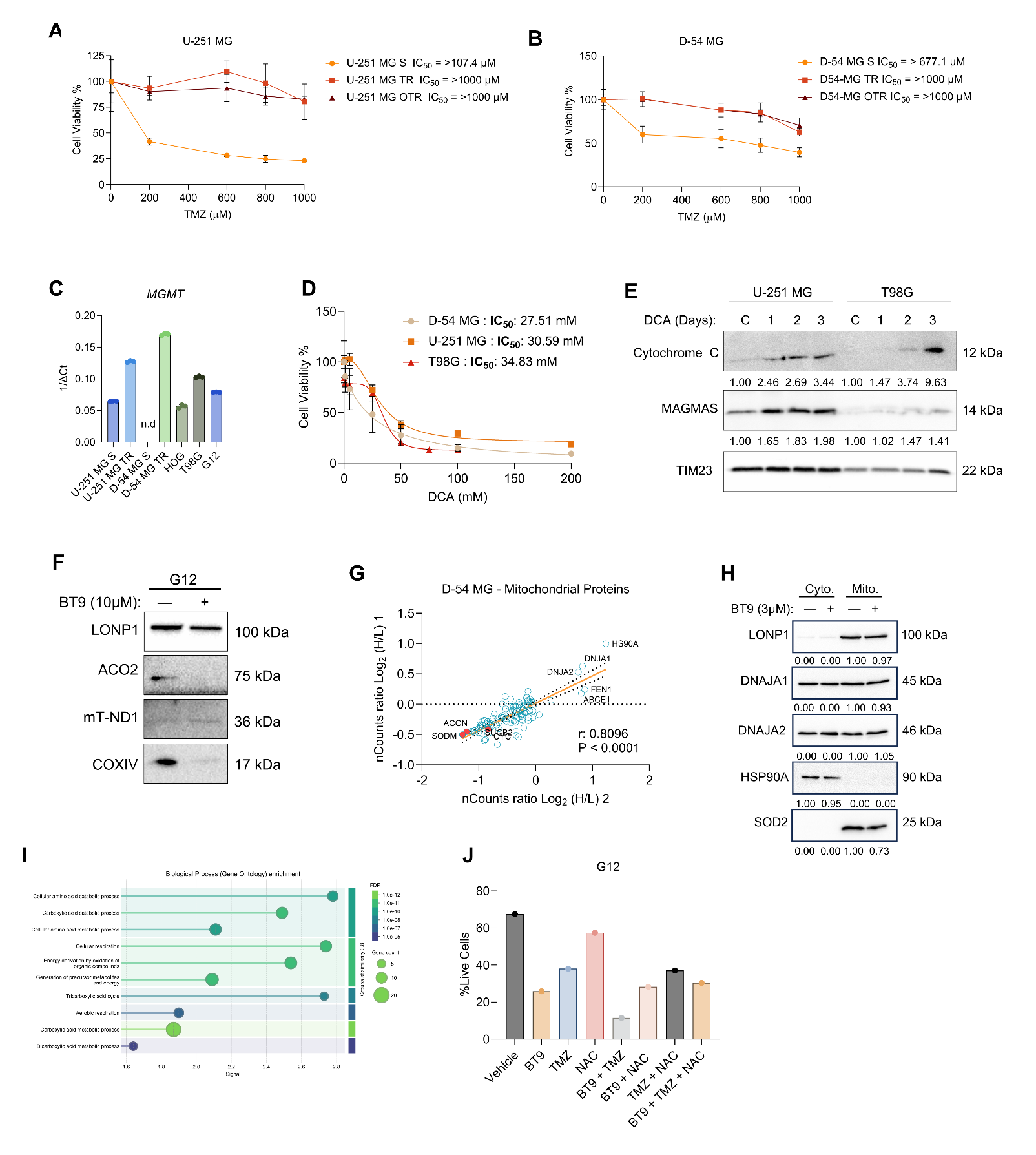


**Supplementary Figure S1**. TMZ resistance in GBM cells and proteomic changes after MAGMAS inhibition. Cell viability of TMZ-resistant GBM lines (A) U-251 MG S, TR, OTR, and (B) D-54 MG S, TR, OTR lines treated with increased doses of TMZ for 5 days and assessed by MTT. (C) *MGMT* mRNA expression levels from different glioma cell lines quantified by qPCR. Data represented was normalized by *ACTB* (delta Ct) and represented as 1/ΔCt for visualization. (D) Cell viability of U-251 MG, D-54 MG, and T98G treated with increasing doses of DCA for 3 days and cell viability was assessed by MTT. The DCA IC_50_ concentration for each respective cell line was selected for subsequent experiments. (E) U-251 MG and T98G were treated with IC_50_ concentrations of DCA (30 mM and 34 mM, respectively) for 3 days. Mitochondrial lysates were harvested and run on an SDS-PAGE gel and transferred to a PVDF membrane. The membrane was immunoblotted against cytochrome C, MAGMAS, and TIM23 (loading control). (F) G12 cells were treated with 10 µM of BT9, and mitochondrial lysates were harvested after 24 hours and blotted for LONP1, ACO2, mt-ND1, and COXIV. (G) Pearson correlation of heavy and light (H/L, L-lysine) peptide counts ratio comparing 2 samples from SILAC labeled D-54 MG cells treated with 3 µM BT9 for 24 hours. Enriched mitochondrial lysates were harvested and processed for analyses using the Thermo Scientific Orbitrap Fusion Lumos Tribid Instrument for peptide detection and quantification. Differential abundances were quantified by calculating H/L ratios for every peptide. (H) Selected protein targets were validated by western blotting from treated mitochondrial lysates. (I) Gene ontology analysis was performed on the most downregulated proteins. (J) Graph depicting % of live G12 cells were treated with 4 µM BT9, 500 µM of TMZ, and/or in combination with 2.5mM of NAC for 3 days. Annexin-V and PI staining was quantified by flow cytometry. Data representative of one independent experiment. All data are presented as mean ± SD.
