# Supplementary material for: MAGMAS Inhibition Enhances Temozolomide Efficacy in Chemotherapy-Resistant Glioblastoma Models
Source: Cancer Res Commun. 2026 Jun 9;6(6):1351–63. doi: 10.1158/2767-9764.CRC-25-0493 (PMC13247981; doi:10.1158/2767-9764.CRC-25-0493)
Supplement: Supplementary Figure S2 — Figure S2. Combinatorial BT9 + TMZ treatment of human primary cell line in orthotopic mouse model. [file crc-25-0493_supplementary_figure_s2_suppsf2.docx]

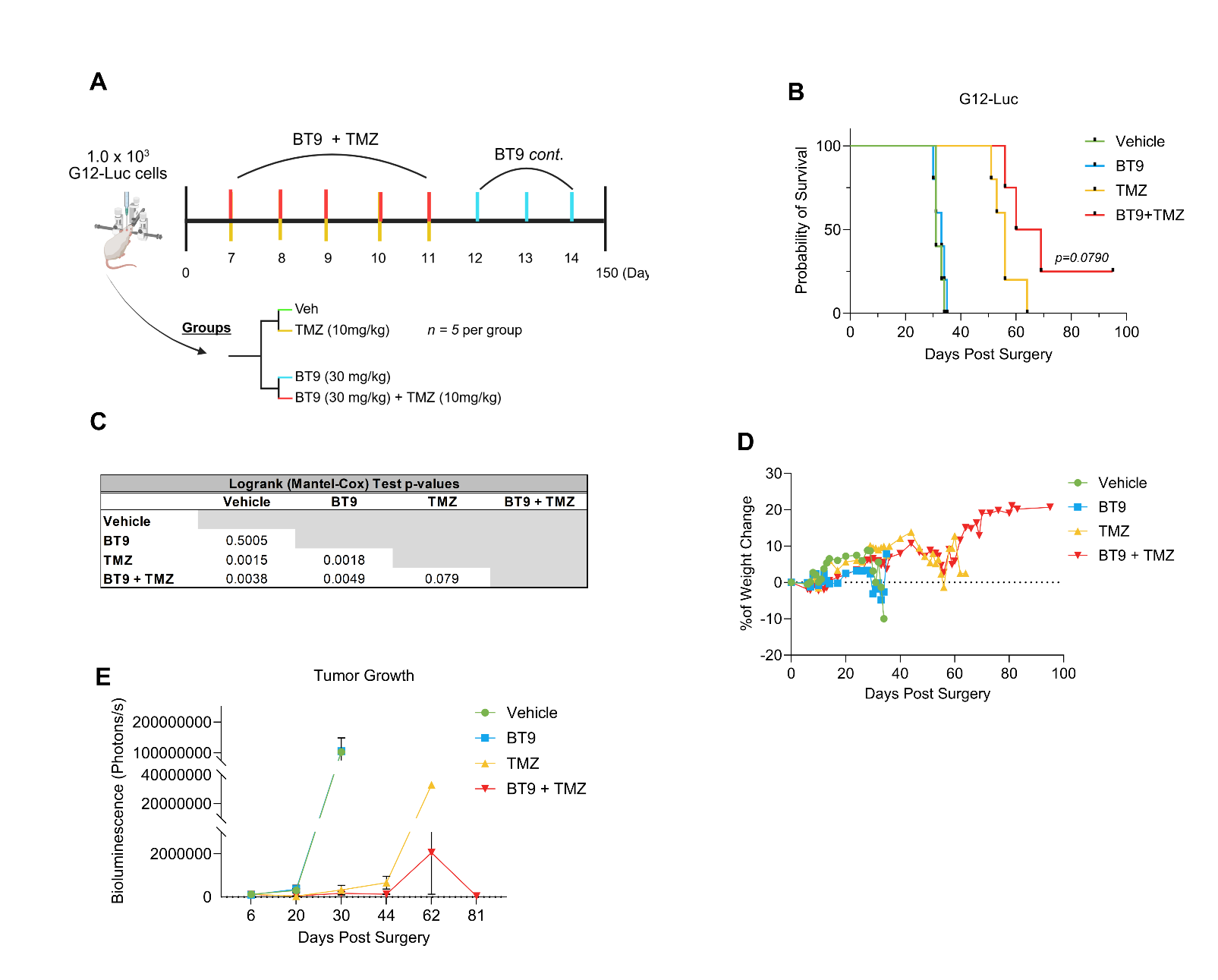


**Supplementary Figure S2.** Combinatorial BT9 + TMZ treatment of human primary cell line in orthotopic mouse model. (A) Timeline schematic of G12-Luc BT9 +TMZ mouse study. Created in BioRender. Lepe, J. (2026) https://BioRender.com/kvudib6 (B) Kaplan Meier curve depicting overall survival of NSG mice transplanted with 1.0 x 10^3^ G12-Luc cells and treated with 30 mg/kg of BT9 for 8 consecutive days by oral gavage and/or 10 mg/kg of TMZ by i.p. for 5 days (*n* = 5 per group) starting 7 days after transplantation. (C) Summary table of the Logrank Mantel-Cox statistical analyses done on the BT9 + TMZ *in vivo* study. (D) Graph depicting % mean changes in weight for each group. (E) Bioluminescence imaging summary (photons/s) of all treatment groups to track tumor growth before and after treatment.
