# Supplementary material for: MAGMAS Inhibition Enhances Temozolomide Efficacy in Chemotherapy-Resistant Glioblastoma Models
Source: Cancer Res Commun. 2026 Jun 9;6(6):1351–63. doi: 10.1158/2767-9764.CRC-25-0493 (PMC13247981; doi:10.1158/2767-9764.CRC-25-0493)
Supplement: Supplementary Figure S3 — Figure S3. Testing of human glioma cells for TMZ sensitivity. [file crc-25-0493_supplementary_figure_s3_suppsf3.docx]

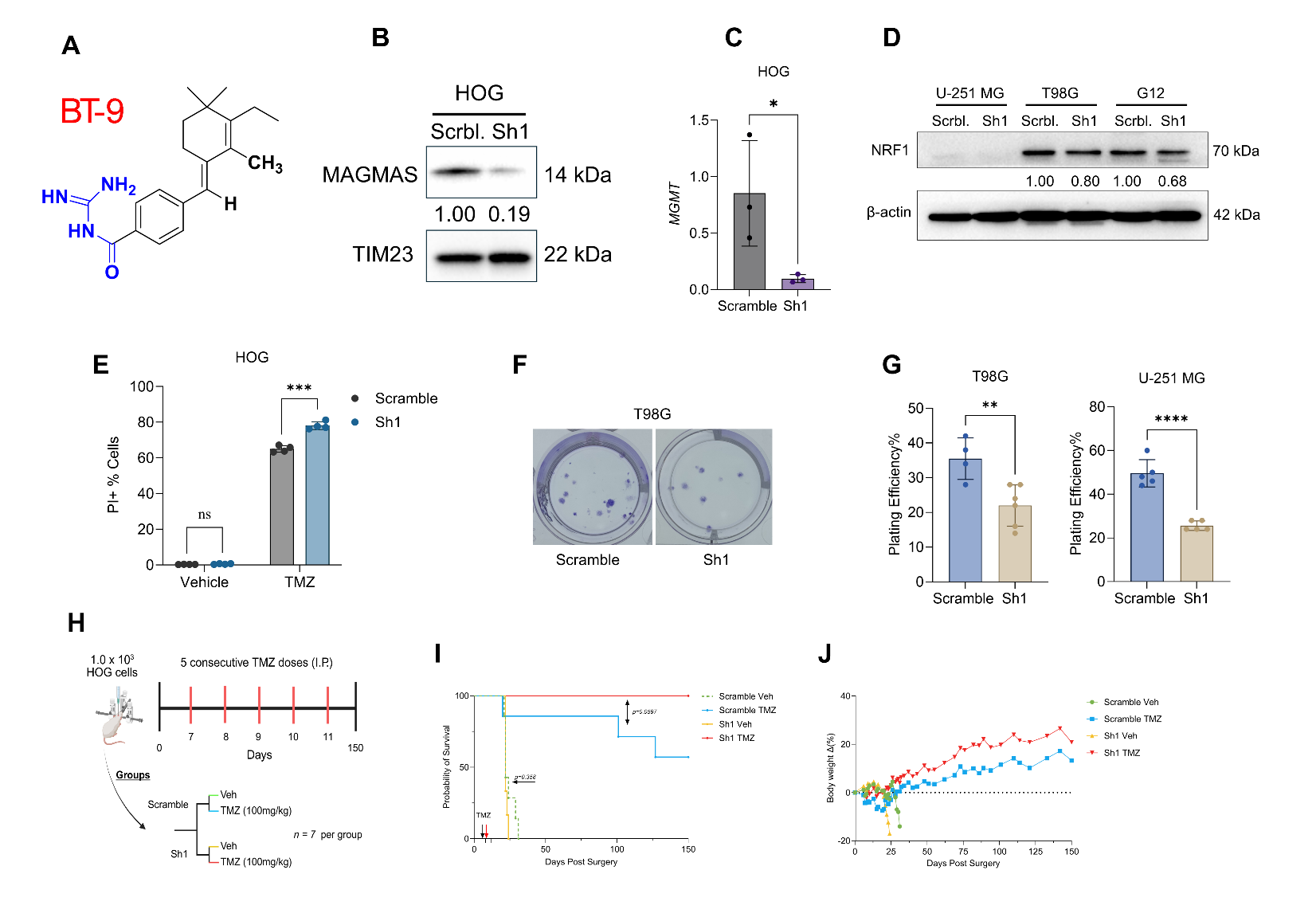


**Supplementary Figure S3**. Testing of human glioma cells for TMZ sensitivity. (A) Chemical structure of BT9. (B) HOG cells were genetically modified to express either scramble or Sh1 (sh*PAM16*). Mitochondrial lysates were harvested from cells and used to verify knockdown efficiency by western blot. (C) RNA was extracted from HOG scramble and Sh1 cells to assess *MGMT* expression levels by qPCR. Statistical analyses were done using a two-tailed, unpaired t-test (*n =* 3). (D) Western blot of U-251 MG, T98G and G12 knockdown whole cell lysates, probed with NRF1 antibody and Β-actin as a loading control. (E) Bar graph of HOG cells, scramble control or Sh1 were treated with a 25 µM concentration of TMZ for 5 days. Significance was determined using a two-way ANOVA, Sidak multiple comparisons test. (F) Representative images of clonogenic assay plates and (G) percent plating efficiency quantification of T98G and U-251 MG scramble control and Sh1 cells. (H) Timeline of HOG cell orthotopic mouse *in vivo* study (*n =* 7 per group). Created in BioRender. Lepe, J. (2026) https://BioRender.com/8wyz7ja. (I) Kaplan Meier curve showing overall survival and p-values were calculated using Logrank Mantel-Cox. Scrbl. veh vs Sh1 veh *p = 0.358,* Scrbl. veh vs Scrbl. TMZ *p = 0.0036*, Scrbl. veh vs Sh1 TMZ *p = 0.0001*, Scrbl. TMZ vs Sh1 veh *p = 0.0055*, Scrbl. TMZ vs Sh1 TMZ *p= 0.0597*, and Sh1 veh vs Sh1 TMZ *p = 0.0002*. Arrows indicate beginning and end of TMZ treatments. (J) Summary graph of % mean change in weight over time for all treatment groups. Data are shown as mean ± SD; * *p* < 0.05, ** *p* < 0.01, *** *p* < 0.001, **** *p* < 0.0001.
